# 3D-bioprinted microenvironments shape the growth and IgG1 monoclonal antibody production of CHO DG44 cells

**DOI:** 10.3389/fbioe.2026.1854326

**Published:** 2026-09-24

**Authors:** Elliott Cowles, Laura Chastagnier, Lucie Essayan, Julia Niemann, Magali Barbaroux, Christophe Marquette, Emma Petiot

**Affiliations:** 1 Université Lyon 1, CNRS, ICBMS, UMR 5246, Villeurbanne, France; 2 Sartorius Stedim Biotech Gmbh, Göttingen, Germany; 3 Sartorius Stedim FMT SAS, Aubagne, France

**Keywords:** biofabrication, bioprinting, bioproduction, Chinese Hamster Ovary (CHO), monoclonal antibodies

## Abstract

**Introduction:**

Three‐dimensional (3D) bioprinting offers new possibilities to recreate in vivo‐like microenvironments for mammalian cells, with potential applications in therapeutic bioproduction.

**Methods:**

In this study, we evaluate the growth, central carbon metabolism, and IgG1 monoclonal antibody (mAb) bioproduction of Chinese hamster ovary (CHO) DG44 cells embedded in 3D‐bioprinted hydrogel constructs under different cultivation strategies: batch, semicontinuous, and both modes preceded by a 3D amplification step in an amplification medium.

**Results and Discussion:**

Across all 3D conditions, CHO DG44 cells exhibited markedly reduced and limited growth over 14 days, most likely due to restricted nutrient diffusion and limited available space imposed by the intrinsic porosity of the bioink. Glucose was not depleted under any condition. In semicontinuous cultures, glucose consumption and lactate production remained approximately constant over the full culture duration, whereas both processes stopped midrun in batch cultures. The relatively low overall glucose consumption indicates that higher cell‐to‐medium ratios could be implemented to improve nutrient utilisation. mAb production closely followed lactate metabolism, with sustained production in semicontinuous cultures and an early plateau in batch mode, resulting in 2‐ to 3‐fold lower final mAb amounts in batch compared with semicontinuous cultures. Introducing a 3D amplification phase more than doubled cumulative mAb production after 14 days. Overall, low growth, low titres, low space–time yields, and residual nutrients in the supernatant show that the current 3D‐bioprinting strategy is not yet optimal for mAb manufacturing. However, despite this apparent weakness of the 3D‐bioprinted environment for production, the 3D specific productivity of 3D‐amplified semicontinuous conditions was found to be higher than the global suspension specific productivity. Consequently, our data suggest that increasing the cell‐to‐ medium ratio and reducing the construct size to mitigate diffusion limitations enhance cell‐specific productivity and process efficiency in 3D‐bioprinted CHO‐ based bioproduction systems.

## 1 Introduction

Monoclonal antibodies (mAbs) have revolutionised modern medicine, playing a central role in immunotherapy to treat cancer, autoimmune disorders, and infectious diseases (Mekala et al., 2024). Their specificity and efficacy have driven a rapidly expanding global market, making them the most present biotherapeutics on the pharmaceuticals market. mAbs are recombinant proteins classically produced by mammalian cell cultures, predominantly Chinese hamster ovary (CHO) cells, cultivated in suspension culture mode. While these classical bioprocesses have been optimised over decades, they face inherent limitations in specific productivity and cellular microenvironment control (Nienow, 2006). The cell culture process generally allows to harvest 1–10 g/L of the drug substance requiring expensive large-scale production up to 25,000 L stirred tanks to manufacture large amounts of antibody (Kelley, 2024).

To cope with these limitations, bioprocess intensification strategies have been actively pursued to meet growing demands and reduce production costs. These strategies include high cell density fed-batch cultures using perfusion of an amplification bioreactor to increase cell concentration during production (MacDonald et al., 2022; Schulze et al., 2022a; Xu et al., 2020), high cell density perfusion cultures (Bielser et al., 2018), media optimisation (Gyorgypal et al., 2025), genetic engineering of cell lines to enhance protein expression (Antony et al., 2024), hydrodynamics optimisation (Wang et al., 2024), and process automation to provide real-time modification of optimal production cellular environments (Schulze et al., 2021). As an example, hydrodynamic optimisation using an alternative bioreactor design is used to reduce cell shear stress as a strategy to improve the cell state in the culture and therefore increase productivity and product quality. Strategies relying on stimulating cell-specific production once high cell concentration is reached have also been employed, either by adding signalling molecules to induce production (Schulze et al., 2022b; Schulze et al., 2022a) or by implementing stress steps (Schellenberg et al., 2022) such as a temperature shift (Becerra et al., 2012; Zhang et al., 2015), pH shift (Hogiri et al., 2018; Liang et al., 2024; Xie et al., 2016), or osmotic shock (Kim et al., 2002; Nasseri et al., 2014).

While most of these efforts focused on pushing the boundaries of suspension cultures, the potential benefits of cultivating CHO cells in adherent conditions within three-dimensional (3D) architectures were largely overlooked. Still, few studies have shown that cultivating production cells in a 3D environment, such as microspheres or macroporous microcarriers, could enhance their recombinant protein-specific productivity. The hypothesis of the potential effect of such a 3D cultivation environment relies on the recovery of physiological conditions of native tissues for such cells, including reduced cell growth, changes in central carbon metabolism, and spatial organisation (Breguet et al., 2007; Tharmalingam et al., 2011; Wong et al., 2007).

Thus, as the effect of 3D cultivation environments on CHO therapeutic protein production remains largely unexplored, we propose evaluating the effect of bioprinting CHO DG44 on monoclonal antibody production. In previous work, we demonstrated that such cells could grow in a 3D-bioprinted biocompatible and proliferative hydrogel (Chastagnier et al., 2025; Marquette et al., 2024a). In such an environment, these cells exhibited *in vivo*-like phenotypes, i.e., reduced growth, better-balanced metabolic activity, and 3D organisation. The present study aims to go further by evaluating bioprinted CHO production capacity to position this 3D culture modality within the panel of tools available for bioproduction. Thus, we have assessed and documented the 3D cell growth, metabolism, and mAbs productivity of 3D encapsulated CHO DG44 under various bioprocess cultivation conditions and at small scale.

## 2 Materials and methods

### 2.1 Cell line and amplification

A recombinant IgG1-secreting CHO DG44 cell line (Cellca-Sartorius) was used for this study. Cells were thawed in stock and adaptation medium (SAM) (4Cell XtraCHO Stock & Adaptation Medium, CQP3FA1114, Sartorius) supplemented with 6 mM GlutaMAX (35050061, GIBCO), centrifuged at 300 *g* for 5 min, and resuspended in warm SAM at 37 °C. The cells were cultivated in shake flasks in an agitated incubator with 7.5% CO_2_ at 130 rpm. CHO DG44 cell amplification consisted in diluting cells every 3–4 days to a concentration of 0.2 × 10^6^ cells/mL. To maintain clone selection, only the three first expansions were performed with medium supplementation with 15 nM methotrexate (M8407, Sigma-Aldrich).

### 2.2 Biofabrication of CHO DG44 constructs

Bioink was composed of three solutions prepared in a calcium-free DMEM (21068028, GIBCO), further denominated DMEM(−), and supplemented with penicillin (15140122, GIBCO)/streptomycin (15290026, GIBCO) antibiotics to a final concentration of 250 U/mL and 5 μg/mL, respectively. The three solutions were prepared the day before bioprinting; consisted of an 8% (w/v) solution of fibrinogen (F8630, Sigma-Aldrich), a 4% (w/v) solution of sodium alginate (A18565, Alfa Aesar), and a 20% (w/v) solution of pork gelatine (G1890, Sigma-Aldrich); and incubated at 37 °C overnight.

Cells to prepare bioink at 10 × 10^6^ cells/mL were harvested by centrifugation and washed with DMEM(−) (Figure 1A). They were then resuspended first in an 8% fibrinogen (m/v) solution. Then, the 4% (m/v) sodium alginate and 20% (m/v) gelatine solutions were added to the cell suspension to reach a formulation containing 2% of fibrinogen (w/v), 2% of alginate (w/v), and 5% of gelatine (w/v). After 10 min incubation at 37 °C, the bioink was transferred to a printing cartridge (Nordson, France) and further incubated for 30–60 min at 21 °C (Marquette et al., 2024a). Bioink cartridges were then fitted with a 410 µm internal diameter and 6.35 mm long nozzle (7018260, Nordson EFD) and loaded on the pneumatic microextrusion printing tool of a BioAssemblyBot 400 bioprinter (Advanced Solutions LifeScience). Cellularised constructs were printed at 21 °C with a pressure of 20–40 psi, a nozzle speed of 10 mm/s, and an acceleration of 750 mm/s^2^. The Computer Assisted Design (CAD) 3D model used for slab printing was designed on TSIM software (Advanced Solutions Life Sciences) and consists of a cuboid structure of 1 × 1 × 0.2 cm^3^. After bioprinting, 3D constructs were immersed for 1 h in a cross-linking solution composed of 10 U/mL thrombin (T4648, Sigma-Aldrich), 1.4% (w/v) calcium chloride (C5670, Sigma-Aldrich), and 0.4% (w/v) transglutaminase (Activa WM, Ajinomoto). After removing the consolidation solution, the constructs were rinsed twice with a 0.9% (w/v) NaCl solution (64,812, Lab. AGUETTANT) for 5 min and were later transferred in 12-well plates, immersed in cell culture medium, and placed in a 37 °C, 7.5% CO_2_ incubator.

**FIGURE 1:**
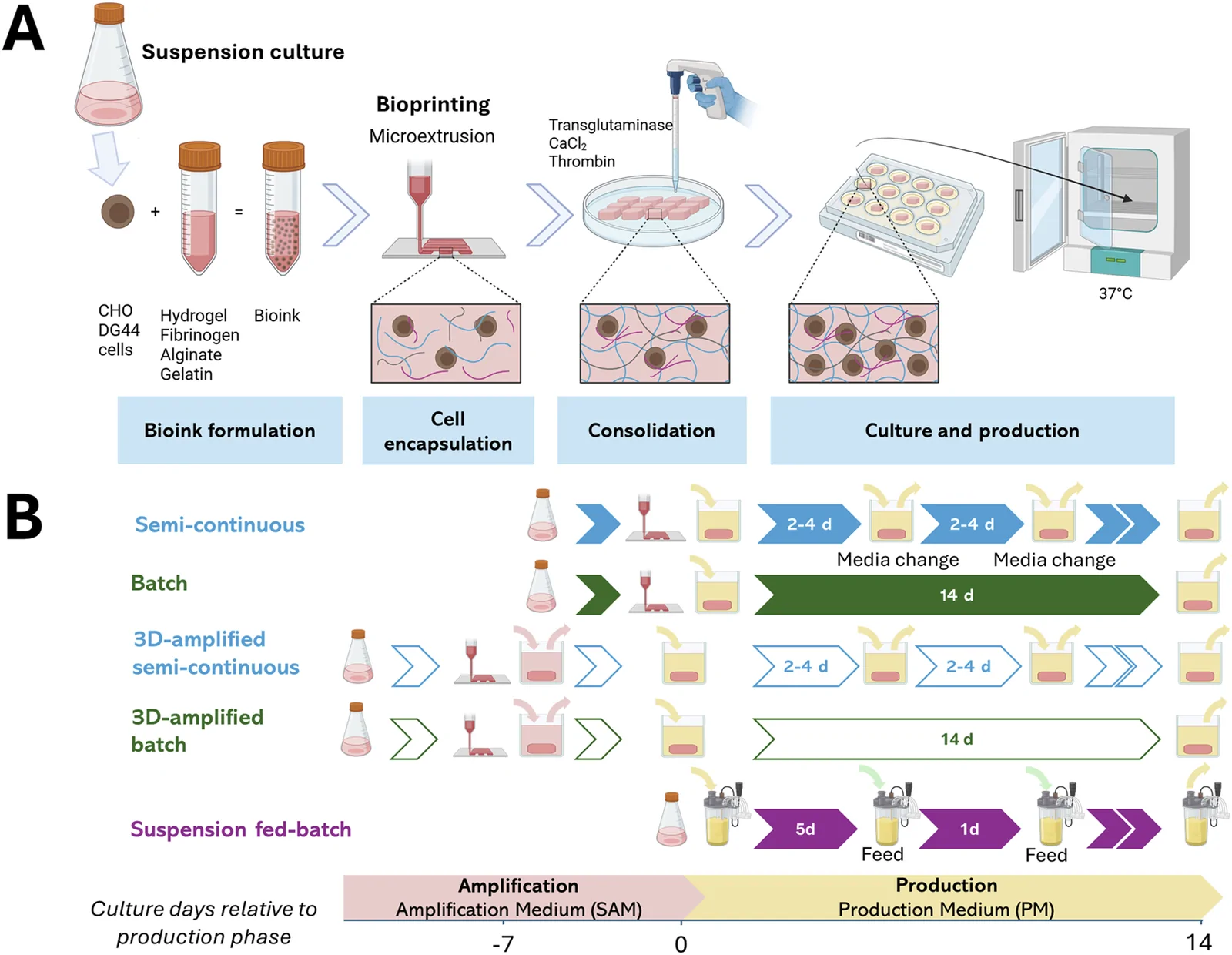
Experimental setup description. **(A)** Workflow for the biofabrication and cultivation of bioprinted constructs seeded with Chinese hamster ovary (CHO) DG44 at 10 × 10^6^ cells/mL of the bioink and **(B)** detailed description of the cultivation condition screened for monoclonal antibody production.

### 2.3 Microextrusion shear stress description and measured effects on CHO DG44

#### 2.3.1 Simulation of shear stress distribution during microextrusion printing

The maximal wall shear stress experienced by the cellularised bioink during microextrusion was estimated *in silico* as previously described (Lemarié et al., 2021). Computational fluid dynamics(CFD) simulations were performed using ANSYS FLUENT® 2020 R^1^ Academic, which applies the finite volume method (FVM). Simulation parameters included a bioink flow rate of 2.35 μL/s, temperature of 21 °C, and nozzle dimensions of 6.35 mm length and 0.41 mm diameter. These data were further processed with the in-house FlowTips® program (Lemarié et al., 2021) using an analytical Poiseuille-based model to calculate the shear rate, shear stress, and axial and radial velocities along the nozzle. Results are reported as theoretical shear stress (Pa) relative to the distance from the nozzle centre. Fitting of the simulation was visualised using MATLAB software.

#### 2.3.2 CHO DG44 cell recovery yield postprinting

To measure CHO DG44 cell recovery after microextrusion and to describe its detrimental effects on the cellularized bioink content, 10 ×10^6^ cells/mL of bioink were loaded into a cartridge and incubated following the standard bioink preparation protocol described in Section 2.2. Bioink samples were extruded through a bioprinting nozzle, weighed, added with at least five times the weighed sample mass of prewarmed DMEM(−) culture medium, and dissolved at 37 °C for 5 min. Dissolved samples were centrifuged for 5 min at 300 g, and cell pellets were rinsed with 500 µL of prewarmed DMEM(−). The collected cell suspension was counted on a Malassez hemocytometer with trypan blue to establish both cell viability and cell yields.

### 2.4 Bioprinted construct culture and monitoring

After cross-linking, bioprinted constructs were dispatched, as described in Figure 1B, to screen feeding strategies. Batch and semicontinuous feeding cultures were immersed in 4Cell XtraCHO Production Medium (PM) (CQP3FA2115, Sartorius) supplemented with 6 mM GlutaMAX (35050061, GIBCO). No medium replacement was performed for the batch condition, while complete medium replacement every 2–4°days was applied for the semicontinuous condition. Similar cultivation protocols apply to 3D-amplified culture conditions with an additional 7-day preamplification step in 4Cell XtraCHO Stock & Adaptation Medium (CQP3FA1114, Sartorius) with complete medium replacement every 2–3°days. Cell growth monitoring was performed by dissociating triplicates of the bioprinted constructs every 2–3°days for cell counting. Sample supernatants were collected for metabolite and mAb quantification. Histological analyses and viability assays via calcein live marker staining was performed on additional samples. Monitoring was conducted on biological triplicates for every 3D condition at every time stamp.

### 2.5 Reference bioreactor CHO DG44 cultivation

A reference culture of CHO DG44 cells in suspension was conducted using a stirred tank bioreactor (Ambr250 HT, Sartorius) following a standard fed-batch process (Schulze et al., 2022b). The CHO DG44 cells were seeded at a concentration of 0.3 × 10^6^ cells/mL in PM. From 5 days of the culture, daily feeding with a concentrated feed solution (4Cell XtraCHO Feed Medium A, CQP3FA3120, SARTORIUS) was applied, complemented with a concentrated amino acid-based feed solution (4Cell XtraCHO Feed Medium B, CQP3FA4119, SARTORIUS) and a concentrated glucose solution (G7528, Sigma) from day 6. The target was to maintain a glucose concentration of 6 g/L throughout the cultivation. Sampling was performed every day for cell growth, metabolite, and mAb production monitoring.

### 2.6 Bioprinted construct cell content and viability qualification

Total cell counting was performed on 3D constructs postdissociation to qualify cell growth kinetics as previously developed by Chastagnier et al. (2025). Bioprinted constructs were weighed and shredded. They were then incubated in 500 µL of a 100 mM, pH 7 sodium citrate solution (71497, Sigma-Aldrich) at 37 °C for 5 min. A 500 μL Phosphate Buffer Saline (PBS)-based solution of 6% (w/v) collagenase A (10103586001, Roche Diagnostics) and 1 mg/mL alginate lyase enzyme (A1603, Sigma-Aldrich) was then added to the tubes on top of the citrate solution, achieving a final concentration of 3% (w/v) collagenase A and 0.5 mg/mL alginate lyase. The tubes were incubated at 37 °C and shaken every 5–10 min until complete dissociation of the hydrogel after 20–30 min. After dissociation, the tubes were centrifuged for 3 min at 190 g, and the cell pellets were resuspended in 1 mL of warm PM cell culture medium. The tubes were then weighed to enable the determination of the cell content per gram of the hydrogel. Cell counting was performed using the trypan blue exclusion method and a Malassez hemocytometer.

### 2.7 Monoclonal antibody secretion

Monoclonal antibody titres in the cultivation supernatants were determined using a luminescent-based protein A enzyme-linked immunosorbent assay (ELISA). Maxisorp 96-well black plates (446471, Thermo Fisher Scientific) were coated overnight at 2 °C–8 °C with a 100 µg/mL solution of recombinant protein A (RPA-100, Repligen) in a pH 9 potassium carbonate buffer (26726.297, Prolabo). The following day, the plates were blocked with 3% (w/v) bovine serum albumin (BSA) in PBS (PBSA) for 1 h at 37 °C. Recombinant IgG1 standards (0–1,000 ng/mL) and appropriately diluted culture supernatants (1:400 for 3D cultures; 1:2,000–5,000 for suspension cultures) were added and incubated for 2 h at 37 °C. After washing, the plates were incubated with alkaline phosphatase-conjugated anti-human IgG antibody (1:10^5^ in PBSA) for 2 h at 37 °C (109-055-003, Jackson ImmunoResearch Laboratories), followed by the addition of a CDP-Star substrate (11685627001, Roche Diagnostics) for 10 min. Chemiluminescence was measured, and IgG1 concentrations were calculated from the standard curve. Measurements were performed in duplicate or triplicate.

### 2.8 Glucose and lactate metabolite quantification

Two soluble metabolites, representative of cell central carbon metabolism efficiency, were quantified in 3D culture supernatants: glucose and lactic acid. Glucose concentration levels in cell culture supernatants were determined using a Glucose GOD-PAP enzymatic detection kit (87109, Biolabo), which relies on glucose quantification by the Trinder method. A solution containing glucose oxidase, peroxidase, and 4-amino-antipyrine in phosphate buffer was mixed with a chloro-4-phenol chromophore reagent and used as a reagent solution. Samples diluted 1:5 and standard solutions of 0.25, 0.5, 0.75, 1, and 1.5 g/L were added to the reagent solution in 96-well plates and incubated at room temperature for 20 min. The glucose concentration was determined by absorbance reading at 500 nm using a standard curve established by measuring the absorbance of standards (G7528, Sigma). Analyses were typically run in duplicate or triplicate.

Lactate concentration levels in cell culture supernatants were determined using an L-Lactic acid (K-LATE, MEGAZYME) detection kit. Samples were diluted 1:5 or 1:10 for suspension cultures and mixed with reagents according to the supplier instructions, and absorbance was read at 340 nm after incubation at 25 °C for 10 min. Analyses were typically run in duplicate or triplicate.

### 2.9 3D CHO DG44 organisation and mAb 3D distribution observation by histology

Bioprinted constructs were sampled for histology analyses. Sample preparation included rinsing in PBS and fixation in a commercial paraformaldehyde-based Antigenfix solution (P0014, Diapath) at least overnight at 2 °C–8 °C. Samples were then washed for 5 min in ultrapure water, and gradual dehydration was performed in 30% (v/v), 50%, and 70% ethanol for 10 min each. Samples were stored in 70% (v/v) ethanol until paraffin processing. Dehydration was completed using an ASP300 (Leica) device. The samples were then embedded in paraffin blocks, sliced with a microtome to 5 µm thick sections, and mounted on microscope slides. Before staining, the sections were deparaffinised and rehydrated by bathing slides twice in toluene and sequentially in 100%, 95%, and 70% ethanol and distilled water for 5 min each.

The sections were then stained following an eosin–haematoxylin colouration kit’s protocol (010263, Diapath). The slides were immersed in haematoxylin (H3401, Vector Laboratories) for 4 min, rinsed 3 times with tap water, and immersed in an activation buffer for a few seconds, followed by immersion in 30% (v/v) ethanol. Finally, the slides were immersed in eosin for 40 s, dehydrated gradually in increasing concentrations of ethanol solutions, and mounted using Biomount DPX (BM500, Biognost). Histology slides were finally scanned using a ZEISS Axioscan 7 scanner.

Immunostaining was also used to detect the presence of monoclonal antibody within the bioprinted slabs. After sample paraffin embedding and slicing into 5 µm sections, the samples were unmasked using a 800-U/mL hyaluronidase solution (R3506, Sigma-Aldrich) in PBS and incubated for 30 min at 37 °C. The slides were washed three times in a PBS bath for 5 min, and then, the sections were blocked with 5% (w/v) BSA (04-100-812C, Euromedex) for 1 h at room temperature. A 1:50 dilution of anti-human goat FITC-conjugated IgG solution (109-095-003, Jackson ImmunoResearch Laboratories) was incubated on the sections for 2 h at 37 °C. The samples were then washed three times in a PBS bath for 5 min in the dark. Finally, nuclei were counterstained using DAPI (300 nM in PBS) for 15 min at room temperature and then washed three times in a PBS bath for 5 min. Image acquisition was then performed using a NIKON ECLIPSE Ts2R inverted microscope.

## 3 Results and discussion

This study aims to evaluate the capabilities of bioprinted CHO DG44 cells to produce therapeutic human IgG1 monoclonal antibody. Cell 3D encapsulation was performed using microextrusion bioprinting technology, a method known to enable the efficient production of large-scale cellularised constructs (Chastagnier et al., 2026; Dufour et al., 2024). Our descriptive study was focused on several cell physiological aspects. First, we aim to confirm CHO DG44 survival during microextrusion and their corresponding cell recovery yield postbioprinting. Second, we described the growth capacity and metabolic activity of bioprinted CHO cells in bioproduction-dedicated media. Then, ultimately, we detailed their monoclonal antibody production capacity. Additionally, to identify the effect of the cell growth phase and cell feeding on mAb production capacity, four culture conditions (Figure 1B) were used to evaluate various bioproduction scenarios. Thus, we tested (i) the addition of a 7-day 3D growing phase prior to mAb bioproduction in a dedicated amplification medium (hereafter referred to as 3D amplification) and (ii) the effect of two feeding modes: semicontinuous (with complete medium replacement every 2–4 days) or batch (using a bolus feeding strategy). Calculations used are shown in Supplementary 1.

### 3.1 Microextrusion bioprinting stress effect on CHO DG44 viability

The microextrusion of cell-laden hydrogel is well known to be a stressful process for cells. These conditions are closely related to the hydrogel’s viscosity, its shear-thinning behaviour, the geometry of the extrusion nozzle, and the applied flow rate as described in our previous detailed experimental study on dermal fibroblasts (Lemarié et al., 2021).

While the selected printing parameters are among the gentlest from previous literature (short nozzle, low flow rate, and shear-thinning bioink), a preliminary understanding of how the microextrusion process affects CHO DG44 cells was still mandatory. Thus, as a first step, an *in silico* description of biomaterial flow and shear stress distribution was performed under our operating conditions. The simulated operating conditions are a flow rate of 2.35 μL/s, a shear-thinning bioink viscosity profile at 21 °C, and a nozzle diameter of 410 µm and length of 6 mm. The simulation of the shear stress in the bioprinting nozzle is shown in Figures 2A,B, demonstrating a gradient of shear from the centre (0) to the wall (205 µm), reaching a maximum of 3,506 Pa·s. This gradient is creating a reduction of the bioink flow rate from the centre to the nozzle wall with 60% of the extruded volume subjected to shear stress rates between 637 and 2,579 Pa·s. Such a descriptive *in silico* analysis should be coupled with the evaluation of cell resistance to mechanical shear stress to identify zones where cells will eventually be mechanically degraded by shear stress.

**FIGURE 2:**
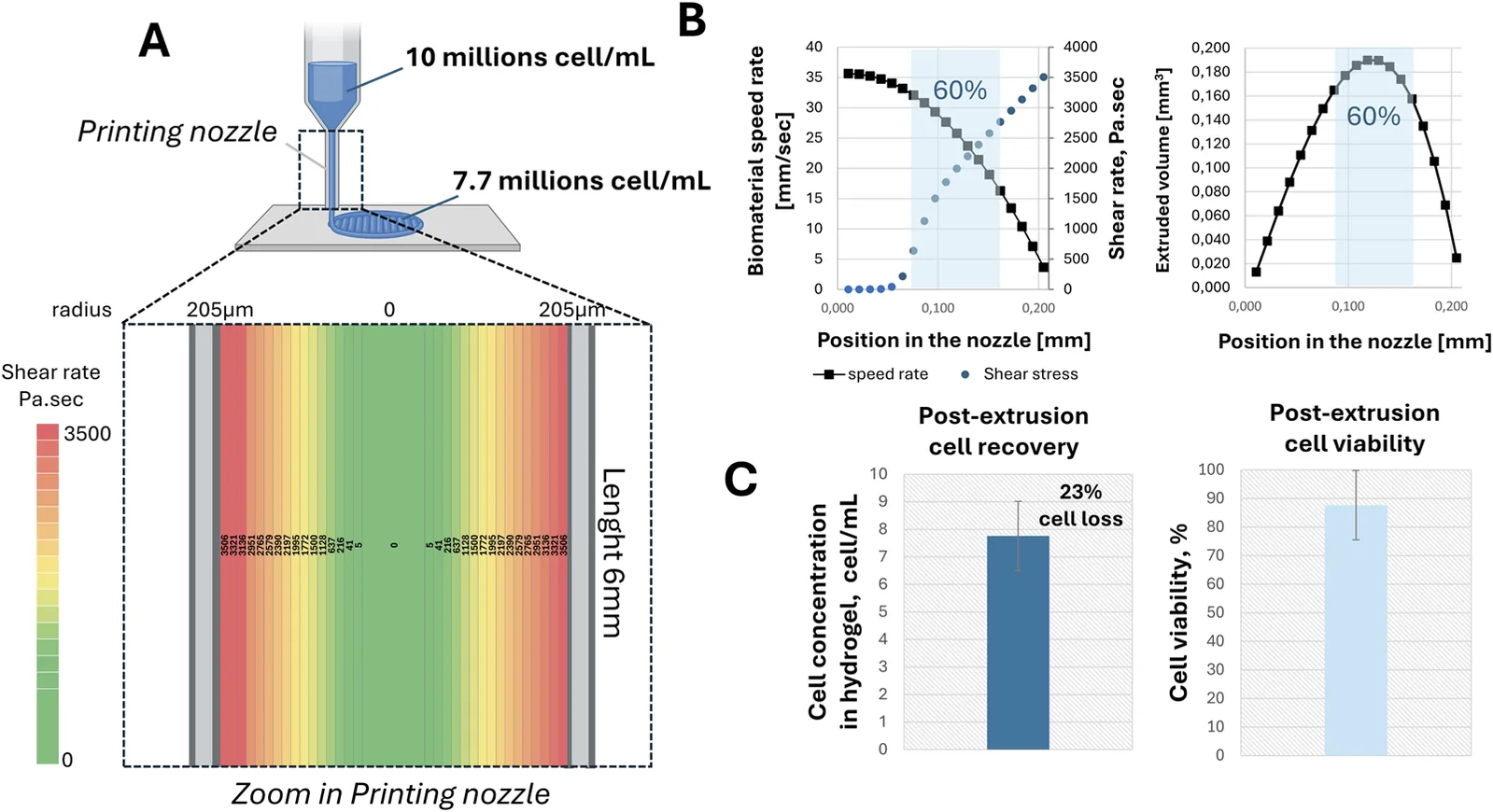
Microextrusion bioprinting shear stress analysis of Chinese hamster ovary (CHO) DG44 cells. **(A)** Simulated syringe model and distribution of wall shear stress in the bioprinting nozzle (calculation parameters: nozzle of 6.35 mm length and 400 µm diameter; flow rate of 2.35 μL/s; temperature 21 °C). **(B)** Distribution of the shear rate (●), biomaterial extrusion rate (■), and volume inside the nozzle (□). **(C)** Measured CHO DG44 cell recovery and viability postprinting on microextruded samples (0.4–1.6 g of bioink per samples). Samples were collected in triplicates.

Thus, regarding the distribution of shear simulated in our bioprinting nozzle, to have a global view of our process effect, it was mandatory to evaluate the cell viability and recovery of CHO DG44 cells during the 3D-bioprinting process prior to the description of cell growth patterns. Experimental determination of cell recovery was performed by measuring cell-laden hydrogel content after microextrusion. This determination was performed under the same condition as the bioprinting experiment, i.e., with a hydrogel loaded with 10 million cells/mL, with a bioink formulation process allowing to reach similar rheology (bioink temperature, flow rate, and viscosity). The bolus of cell-laden hydrogel was then extruded through the bioprinting nozzle and weighed. The cells within the hydrogel bolus were analysed after dissolving the bioink in prewarmed medium. This step was followed by manual cell counting using trypan blue to distinguish live from dead cells. The evaluated cell recovery rate demonstrated a cell loss of 23% ± 6.3% for loaded cells, with a recovered cell viability of 92% ± 5% (Figure 2C). These results are in the high range of the studies describing the effect of microextrusion on cell viability, while very few precisely describe the effective cell recovery per se (Müller et al., 2023).

### 3.2 CHO DG44 3D organisation and cell growth in different cultivation mode

To understand CHO DG44 cell productivity behaviour in a 3D environment, it is crucial to describe cell growth and metabolic behaviour. Preliminary work has stated that bioprinted CHO DG44 cells were able to grow as aggregates in 3D hydrogels, using a culture media (DMEM supplemented with 10% Foetal bovine Serum (FBS)) not optimised for bioproduction (Chastagnier et al., 2025). In the present study, to evaluate the monoclonal antibody bioproduction capacity of CHO DG44 cells, we investigated their behaviour, including growth profiles and 3D organisation dynamics, using our bioproduction culture protocols and specific culture media. Thus, as depicted in Figure 1B, four bioproduction scenarios were implemented on a 3D-bioprinted CHO DG44 production system. Two of these scenarios included a 3D-amplified cell phase before the bioproduction and two feeding strategies were tested semicontinuous or batch feeding. In the case of the 3D-amplified scenarios, a specially designed amplification culture medium was used during the first 7 days of the culture before being replaced by a production-dedicated medium for the following 14 days.

#### 3.2.1 Cell growth kinetics

Growth kinetics was qualified by the dissociation of constructs every 2–6 days to isolate and count cells, enabling the calculation of cell concentration in the hydrogel at each time point. The cell concentration determined at day 1 was 4.5 × 10^6^ ± 1.1 × 10^6^ cells/g, whereas the expected value was 7.7 × 10^6^ cells/g according to the cell recovery study performed after microextrusion (Section 3.1; Figure 2C). Such a 40% drop in the real measured cell content after 1 day of the culture is consistent with visual observations of live and dead cells performed on day 1 (see Supplementary 2). Such an additional loss in viable cells could be attributed to both (i) poor cell recovery to shear stress applied during printing and (ii) effect of the consolidation solution. Such an effect has already been described in a previous study where solution osmolarity was demonstrated to strongly affect cell viability postprinting (Chastagnier et al., 2025).

It is also notable that the construct dissociation process exhibits 25% variability in final cell counts. Having acknowledged this bias, construct dissociation was used to monitor cell concentrations over the course of the culture and to describe CHO DG44 growth trends under the different bioproduction scenarios tested (Figure 3A).

**FIGURE 3:**
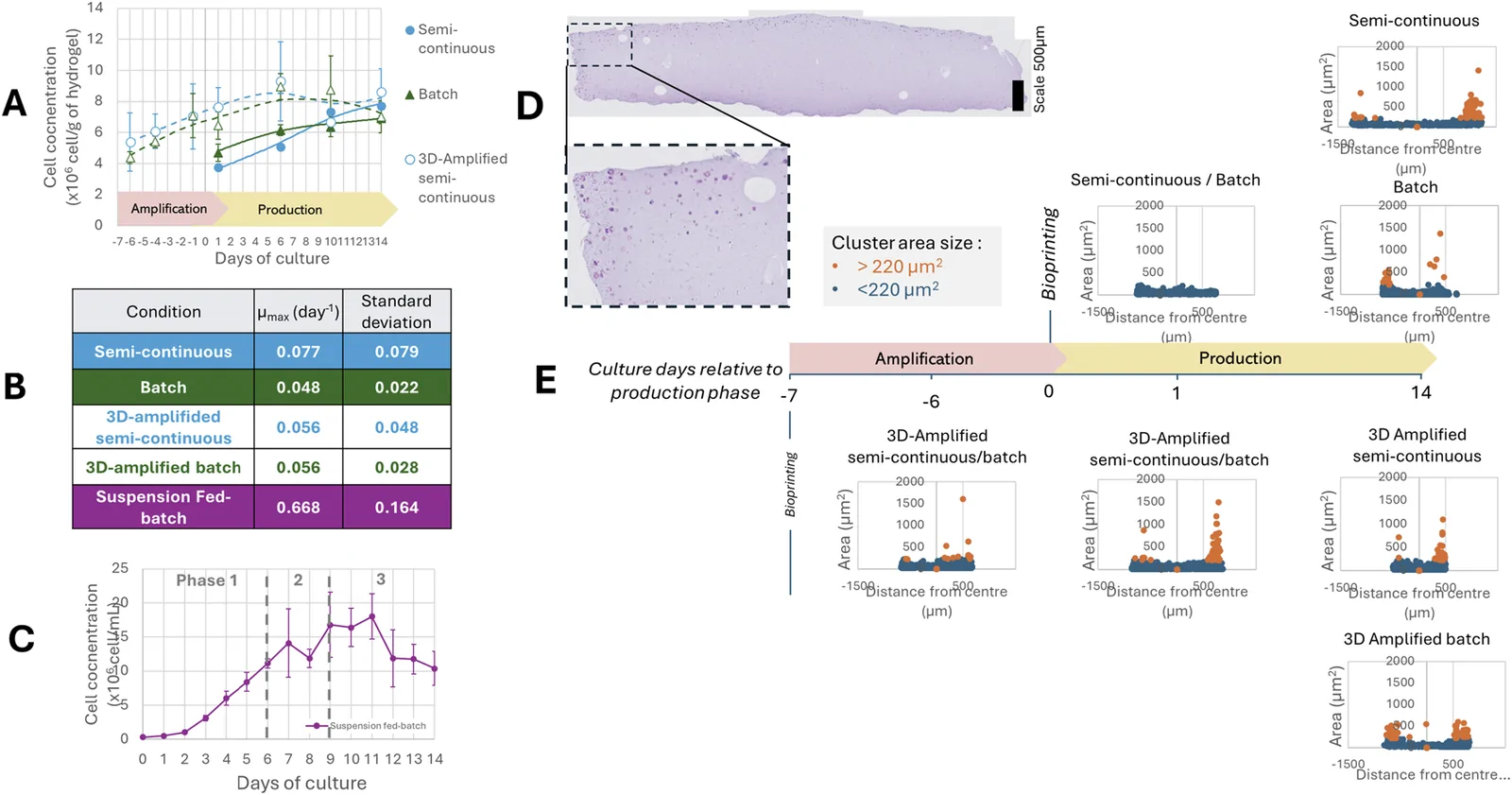
Growth and architecture of three-dimensional (3D) bioprinted cells. **(A)** Growth profiles of Chinese hamster ovary (CHO) DG44 cells in bioprinted constructs; values are expressed in cells per mass of hydrogel after enzymatic dissociation of constructs. **(B)** Maximum growth rates of CHO cells in bioprinted constructs and suspension culture. **(C)** Growth profiles of the CHO DG44 cells in a suspension bioreactor culture. **(D)** Haematoxylin–eosin-stained histology slide of a transversal cut of a bioprinted construct. **(E)** Distribution of cell cluster sizes inside of a section of the bioprinted construct at days 1 and 14 of the production phase for semicontinuous and batch culture conditions and at days 6 (after the first day of the amplification phase) and 1 and 14 of the production phase for 3D-amplified conditions.

First, we observed that both the amplification and production media used supported the 3D cell growth of CHO DG44 (see Figure 3A). Growth rates in the first 5 days of each condition were equivalent and were characterised by a linear growth phase with a mean specific growth rate of 0.059 ± 0.012 days^−1^ (Figure 3B).

Following this first culture phase, the cell concentrations continuously increased over the culture under all conditions up to 14 days either in production medium (standard scenarios) or 7 days of amplification medium followed by production medium (3D-amplified scenarios). Thus, all constructs exhibited similar cell concentrations after 14 days of the culture, reaching 8.2 × 10^6^ ± 1.6 × 10^6^ cells/g, corresponding to a 1.8-fold expansion.

For scenarios involving a 7-day 3D cell pre-amplification phase (3D amplified conditions), cell concentrations plateaued after 14 days of culture, subsequently decreasing until the end of the culture period. This phenomenon happened both under batch and semicontinuous conditions; thus, involving the 3D cultivation system inherently implies cell growth limitation.

For comparison and as a control for cell quality, CHO DG44 cells from the same seed lot were cultivated as a suspension in fed-batch mode from day 5, and their growth trend was monitored. As previously described in several papers (Müller et al., 2022; Schulze et al., 2022a), CHO cells exhibited a very different behaviour in suspension, with an exponential phase over the first 6 days of the culture from 0.3 × 10^6^ cells/mL to 11 × 10^6^ cells/mL, followed by a plateau with a final 60-fold expansion reaching 18 × 10^6^ cells/mL at day 11. Such a growth behaviour was characterised by a 10-fold higher maximum growth rate than in 3D cultures (Figures 3B,C).

Thus, under our experimental conditions, we first confirmed behaviour similar to that already observed in a previous study of 3D-bioprinted CHO, with lower cell growth in 3D than in the suspension culture (Chastagnier et al., 2025). Interestingly, in the present case, our growth rate is much lower than that in previous work (0.059 days^−1^ vs. 0.29 days^−1^, respectively); however, the lag phase observed previously is no longer visible, as our cells grow linearly from the very beginning of the culture. This could be attributed to better matching the medium content to CHO cell needs. In our previous work, a suboptimal medium DMEM + 10% FBS was used, while both the amplification and production media selected for this study are dedicated to CHO cell bioprocesses.

Still, CHO DG44 cells cultivated in semicontinuous mode with regular feeding show neither a remarkably higher growth rate than their batch equivalents nor an increased maximal cell concentration. This observation is surprising, since in the literature (MacDonald et al., 2022), semicontinuous or perfusion cultures are known to enable extended cell growth by replenishing nutrients and removing metabolic waste products. The present results suggest that growth limitation in a 3D cell-encapsulated culture is not related to nutrient limitation or the accumulation of waste products in the supernatant (presented in Section 3.3), but rather to other mechanisms. In previous studies, CHO cells grown in alginate/poly-L-lysine hydrogel microbeads or on macroporous microcarriers also showed limited maximum cell concentrations, which the authors linked to limited space availability in the porosity of the 3D structures (Breguet et al., 2007; Tharmalingam et al., 2011). Additionally, the microporosity of the present hydrogel has already been identified as an important parameter driving the proliferation of other embedded cell lines (Chastagnier et al., 2025).

#### 3.2.2 Cell distribution and 3D organisation

To identify cell distribution and organisation within the 3D-bioprinted constructs, histological slices were stained, imaged, and analysed to quantify cellular clusters at different time points. Figure 3D presents a selected image of a bioprinted construct section to illustrate typical cluster distribution, and Figure 3E presents the quantitative analysis of the clusters, plotting clusters’ diameter as a function of their position within the entire constructs. This analysis allowed to confirm that at day 1 postprinting, single cells were homogeneously distributed within the structure (Figure 3E). Most cluster areas identified at day 1 were less than 220 μm^2^, corresponding to theoretical round-shaped cells of 16.7 µm in diameter. This diameter is in complete agreement with the previously published CHO DG44 cell mean diameter of 15.75 µm, which was determined in a suspension cell culture (Schulze et al., 2022b).

Then, along the culture, as illustrated in Figure 3E, CHO DG44 clusters were growing, reaching multicellular cluster areas of up to 1,500 μm^2^ after 8 days of the culture (day 1 of production for 3D-amplified conditions). In a highly reproducible manner and regardless of the four bioproduction scenarios implemented, cluster growth appears only at the periphery of the constructs within an apparent maximal development depth of approximately 250 μm, thereby creating a gradient of hydrogel cellularity. Such gradients have been observed in similar bioprinted models with production cell lines, cancer-associated fibroblasts, and colorectal cancer HT29 cells (Chastagnier et al., 2025; Marquette et al., 2024b). The inability of clusters to grow beyond 250 µm suggests that nutrient and/or oxygen transport to the construct core is limited, most likely due to slow solute diffusion through microporous hydrogels.

Thus, we identified two distinct effects of embedding CHO DG44 cells within a 3D hydrogel that directly affect their growth: (i) limited available space for cellular development within the gel and (ii) restricted nutrient transport resulting from the low diffusion coefficients of molecules within thick hydrogels. Interestingly, cell growth quantified in Section 3.1 is happening in only 35% of the slab volume. This allows us to presume we obtain a local cell amplification factor in the slab periphery region closer to 5.6, thereby reaching estimated cell concentrations of 25.3 × 10^6^ cells/g of hydrogel. Such analyses permit us to consider that growing CHO DG44 cells in structures allowing a higher media/hydrogel interface, such as the macroporous structures already presented by Chastagnier et al. (2023), or small embedding systems such as hydrogel microbeads of 100–300 μm, or even more microporous hydrogels produced through reduced cross-linking as suggested by Chastagnier et al. (2025), would allow us to recover cell densities similar to those of suspension cultures.

### 3.3 Effect of bioproduction scenarios on the central carbon metabolic activity of 3D CHO cells

CHO DG44 central metabolism activity was evaluated by quantifying glucose and lactate concentrations in culture supernatants collected at different time points. From these measurements, cumulative glucose consumption (Figure 4A), glucose concentration profiles (Figure 4B), cumulative lactate production (Figure 4C), and lactate concentration profiles (Figure 4D) were established.

**FIGURE 4:**
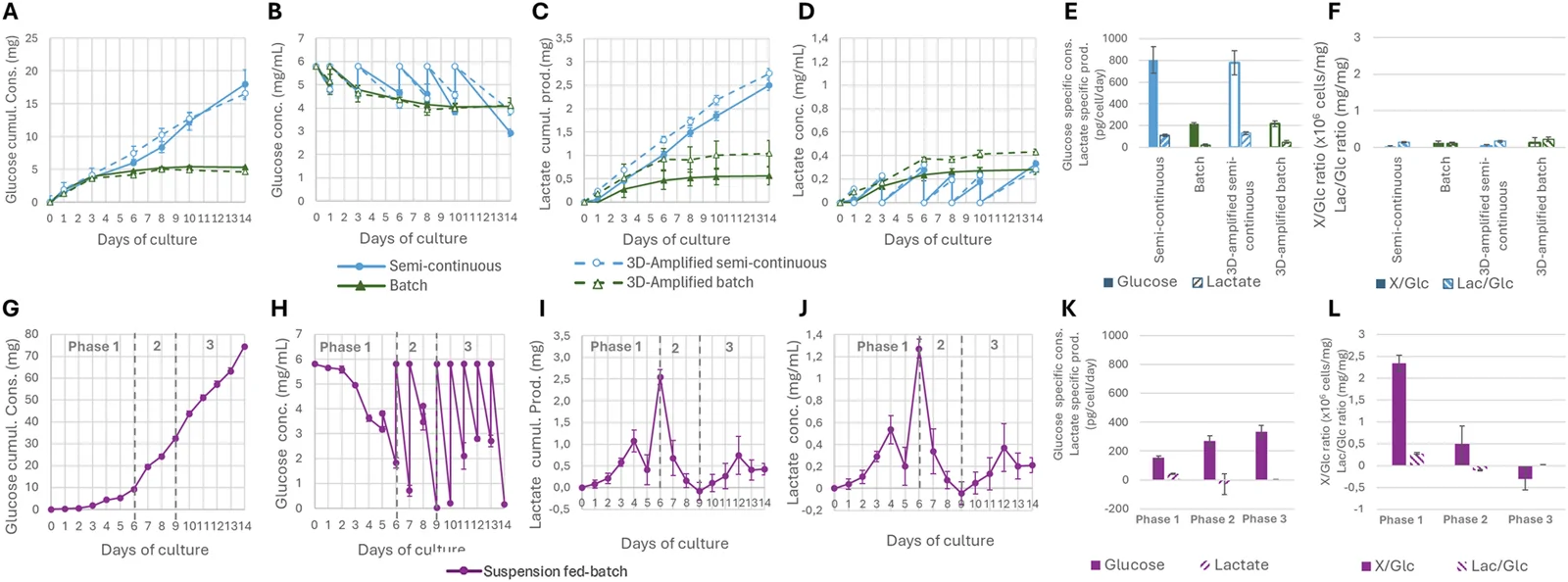
Description of Chinese hamster ovary (CHO) cell metabolism. **(A)** Evolution of glucose consumption in three-dimensional (3D) culture supernatants. **(B)** Evolution of glucose concentration in 3D culture supernatants. **(C)** Evolution of lactate production in 3D culture supernatants. **(D)** Evolution of lactate concentration in 3D culture supernatants. **(E)** Glucose-specific consumption and lactate-specific production in 3D culture supernatants. **(F)** Number of cells generated per mass of glucose consumed and mass of lactate produced per mass of glucose consumed in 3D culture supernatants. **(G)** Evolution of glucose consumption in the suspension culture. **(H)** Evolution of glucose concentration in the suspension culture. **(I)** Evolution of lactate production in the suspension culture. **(J)** Evolution of lactate concentration in the suspension culture. **(K)** Glucose-specific consumption and lactate-specific production in the three phases of the suspension culture. **(L)** Number of cells generated per mass of glucose consumed and mass of lactate produced per mass of glucose consumed in the three phases of the suspension culture. Glc, glucose; Lac, lactate.

By observing glucose and lactate concentration profiles and associated cumulative consumption/production, two culture mode-related behaviours can be described.

First, under both batch conditions (3D amplified and not 3D amplified), glucose was progressively consumed, declining from an initial 5.8 mg/mL to 4.0 ± 0.2 mg/mL, while lactate gradually accumulated to 0.3 ± 0.06 mg/mL during the first 8 days of the production phase. After 8 days, cells stopped consuming glucose and producing lactate. Interestingly, although glucose-based metabolic activity declined, batch conditions demonstrated persistent cell growth after day 8 of the culture, indicating that 3D-cultivated cells may have a secondary carbon source for cell division. Such behaviour is not typical of CHO DG44 cells, which usually consume their medium glucose content entirely, requiring regular feeding in fed-batch processes (Schulze et al., 2022a). We hypothesise that, in a culture of CHO DG44 embedded in a hydrogel, the local glucose concentration in the gel is too low due to its slow diffusion and that other central carbon metabolism inputs, such as glutamine (GlutaMAX), might replace glucose as the primary carbon source to support cell growth.

In contrast, under semicontinuous conditions, glucose was consumed at a constant rate of 1.2 mg/day over 14 days of the culture (16.8 ± 3.2 mg of glucose consumed in 14 days). This is a consequence of the periodic replenishment of glucose in the culture media. Such glucose consumption is approximately three times higher than that observed under batch conditions (5.3 ± 0.2 mg of glucose consumed in 14 days). In parallel, under both semicontinuous conditions, the cumulative lactate production increased at a constant rate of approximately 0.2 mg/day, finally reaching 2.7 ± 0.2 mg on day 14. Still, lactic acid concentrations were maintained under all conditions below 0.4 mg/mL, which is known to be below the lactic acid inhibitory concentration (Hartley et al., 2018).

CHO DG44 cells are known to modulate their metabolism to consume lactate alongside glucose in the suspension culture when reaching such inhibition ranges, as is clearly visible in Figures 4I,J. This metabolic switch can be described in three phases in both batch and semicontinuous suspension processes: a first phase where glucose is consumed while lactate is produced during exponential growth, reaching high concentrations of lactic acid >1 g/L; a second phase where glucose and lactate are coconsumed in the late stage of exponential growth; and a third phase where glucose is consumed with mild lactate production during cell plateau (Reinhart et al., 2015; Schulze et al., 2022b). Such lactate consumption was not observed at a global level in the supernatant of our 3D culture of CHO DG44 cells. However, one should expect that local 3D lactic acid distribution could provide an environment that leads to such behaviour. This further explains the apparent cell growth of CHO cells observed between 8 and 14 days of the culture without glucose consumption.

To further describe differences between the bioproduction scenarios, glucose-specific consumption and lactate-specific production rates (pg/cell/day) were calculated for each culture condition and are presented in Figure 4E. Semicontinuous conditions showed a 2-to-4-fold higher specific lactate production than batch conditions. Similar trends can be observed in specific glucose consumption, with a 4-fold higher rate in semicontinuous conditions than in batch conditions, emphasising the higher metabolic activity observed in semicontinuous conditions.

Comparing these results with suspension culture behaviour (Figure 4K), the specific glucose and lactate consumption rates observed in both batch conditions most closely resemble the exponential phase of the suspension culture, with 215 pg/cell/day of consumed glucose and 36 pg/cell/day of produced lactate. In contrast, the metabolic rates observed in semicontinuous mode are strongly increased for glucose only and are at least twice as high as the metabolic activity observed in suspension.

Metabolism efficiency was also described using metabolic yields Y_X/Glc_ and Y_Lac/Glc_. As expected, the number of cells generated per glucose consumed (0.15 × 10^6^ cells/mg) for each 3D condition was lower than during the expansion phase of the suspension culture (2 × 10^6^ cells/mg). This again reflects the low growth-oriented metabolism in CHO DG44 3D cultures. Lactate production per glucose consumed under all 3D conditions was found to be similar, ranging from 0.10 to 0.22 mg lactate produced per mg glucose consumed and equivalent to suspension phase 1 (0.24 mg lactate produced per mg glucose consumed) (Figures 4E,F,K,L). Thus, the metabolic efficiency of embedded CHO cells is not affected by either the 3D cell environment or semicontinuous feeding.

Again, we observed that despite enhanced glucose and lactate metabolism under semicontinuous feeding, this did not better support cell growth, most likely due to space constraints as already discussed in Section 3.2.

### 3.4 Evaluation of mAb bioproduction using 3D-bioprinted CHO DG44

The evaluation of the bioproduction capacity of 3D-cultivated CHO DG44 cells was performed by quantifying mAbs released into cell culture supernatants every 2–4 days using an ELISA. Cumulative productions and mAb titres are presented in Figures 5A,B, respectively.

**FIGURE 5:**
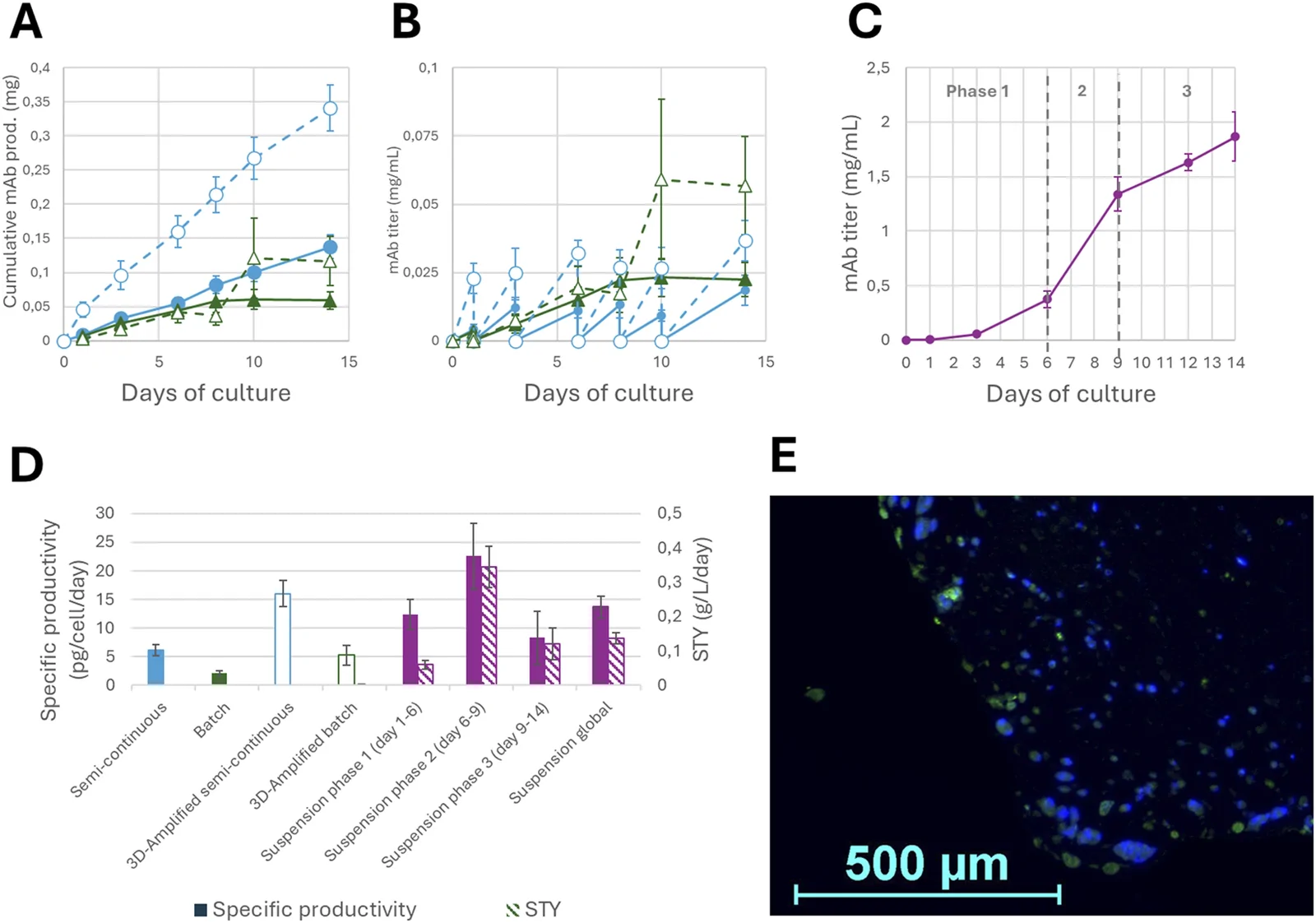
Description of Chinese hamster ovary (CHO) DG44 cell monoclonal antibody (mAb) production. **(A)** Evolution of monoclonal antibody production in three-dimensional (3D)‐bioprinted construct supernatant, **(B)** evolution of mAb titre in the 3D‐bioprinted construct supernatant, **(C)** evolution of mAb production in a 2 mL supernatant in the suspension culture, **(D)** evolution of mAb titre in the suspension cell culture supernatant, mAb‐specific productivity and STY, and **(E)** histology slide of a semicontinuous condition 3D‐bioprinted construct at day 14 of production, IgG immunostaining (green), and nuclei DAPI staining (blue).

First, all the 3D bioproduction scenarios enabled the production of detectable mAb concentrations with titres of 0.02–0.06 mg/mL and cumulative productions ranging from 0.06 to 0.35 mg.

Within both batch 3D conditions, the mAb production profile was highly similar to the cumulative glucose consumption and lactate production profiles, characterised by a constant increase during the first 8 days of the culture followed by a stationary phase, thus indicating a coupling of metabolic and bioproduction cellular activities in these cases. Batch yielded the lowest cumulative production at day 14 with 0.06 ± 0.01 mg (Figure 5A), while the addition of a 3D amplification phase allowed doubling production up to 0.12 ± 0.04 mg.

Both semicontinuous and 3D-amplified semicontinuous conditions yielded higher mAb amounts than their respective batch counterparts, reaching 0.14 ± 0.02 mg and 0.34 ± 0.03 mg at day 14, respectively. This indicates that a continuous feeding strategy, even without affecting cell growth, might influence production. Here again, the addition of a 7-day 3D amplification step doubled mAb production to 0.34 ± 0.03 mg.

Comparing specific productivities (Figure 5D) enabled the identification of similar differences between culture conditions. Batch conditions had the lowest specific productivity (2.1 ± 0.5 pg/cell/day). A positive effect of the 3D amplification step was evident, enabling it to reach 5.3 ± 1.8 pg/cell/day. Also, as suggested earlier, semicontinuous conditions were the most productive with 16.0 ± 2.3 pg/cell/day and 6.1 ± 1.0 pg/cell/day for the conditions with and without 3D amplification, respectively.

Looking at these production dynamics, in 3D-amplified bioproduction scenarios (Figure 2A), the batch culture stationary phase from day 10 was associated with zero productivity. On the contrary, under 3D-amplified semicontinuous conditions, when cells reached a stationary growth phase on day 6, the production rates remained constant (Figure 5A). These opposed observations suggest that the stationary phase reached under batch or semicontinuous conditions permits obtaining cells in different physiological states, which can strongly affect the mAb production dynamics. The only remarkable differences between those two behaviours are that glucose feeding and consumption are constantly maintained in semicontinuous mode, while lactate concentrations periodically drop to zero. As in standard perfusion bioproduction processes (Liang et al., 2023), this could help preserve cells in an optimal physiological state for production.

To benchmark such production capacities, the production dynamics and capabilities of our CHO DG44 cell line were assessed in a fed-batch suspension culture. As the biomass available for production was remarkably higher in the suspension culture, the associated production was, as expected, 11 times higher than that in the 3D cultures. The suspension culture reached a titre of 1.9 ± 0.2 mg/mL after 14 days with a global specific productivity of 13.7 ± 1.9 pg/cell/day (Figure 5C)—representing 3.7 ± 0.4 mg in 2 mL of supernatants—to facilitate comparison with 2-mL well plate cultures in 3D culture scenarios. mAb production in the suspension culture was not constant but rather composed of three successive phases, with a maximal specific productivity of 22.5 ± 5.7 pg/cell/day at the end of the exponential growth phase (Phase 2, Figure 5D). This is a typical trend for the CHO suspension culture (Schulze et al., 2022a), which is strikingly similar to the three phases of growth described in Figure 4.

Comparing 3D and suspension production results side by side highlights an 11-fold lower production for the best 3D condition (3D amplified and semicontinuous) compared with suspension. This production led to a 33-fold higher titre in the suspension culture than in the best-performing 3D culture. Despite this apparent weakness of the 3D-bioprinted environment for production, the 3D specific productivity of the 3D-amplified semicontinuous conditions was found to be higher than the global suspension specific productivity (Figure 5D).

Furthermore, space–time yield (STY) calculations helped us position the 3D culture in terms of process efficiency. Although semicontinuous conditions produced three times more antibody than their batch equivalents, they also require three times more medium volume. STY was therefore comparable between the batch and semicontinuous conditions. Nevertheless, 3D amplification only doubled their STY, suggesting that 3D amplification increases global process efficiency. It is, however, essential to note that even though specific productivity in 3D-amplified semicontinuous conditions was higher than in the global particular suspension productivity culture, the STY of the suspension culture remained around 170 times higher than any of the present 3D cultures, yielding 0.35 and 0.002 g/L/day for the suspension and 3D-amplified semicontinuous conditions, respectively (Figure 5D) (Müller et al., 2022).

Finally, to completely characterise the 3D production system, we investigated the potential presence of secreted mAbs within the 3D hydrogel. This sequestration could introduce bias into production determination because all mAb detection was performed in the culture supernatant. On day 14 of production, 3D-bioprinted constructs were harvested, fixed, and sectioned to enable immunohistological characterisation. Staining was performed after fixation with paraformaldehyde, which induces covalent cross-linking between macromolecules within the constructs and embedded cells, including bound antibodies. Consequently, antibodies are expected to remain immobilised within the samples and should not be removed during the subsequent washing steps of the histological procedures. Figure 5E presents a fluorescent microscopy view after mAb-specific immunostaining. No significant fluorescent signal could be found within the hydrogel surrounding cells (stained with DAPI), indicating that most of the produced mAbs are secreted in the medium. However, the colocalisation of mAbs with cell clusters raises the possibility that they were produced but not yet released from the gel or cytoplasm, or that they were sequestered.

In both cases, this might influence the recovery of the mAbs from a 3D production system. One last observation from this histological characterisation is that cells embedded farther than the apparent maximal development depth of 250 µm were not colocalised with mAb staining, suggesting that the nonproliferative cells at the core of the constructs were also nonproductive. Once again, this assumes that the reduction of dead zones by increasing the hydrogel/media interface might be beneficial to overall IgG productivity. Finally, in an attempt to quantify mAbs potentially trapped within the hydrogel, ELISA was performed on the dissociation supernatants from cell counting to measure residual mAb concentrations. These results presented in Supplementary 2 are consistent with the histological analyses indicating little to no mAb content in the hydrogel.

## 4 Conclusion

This work provides the first comprehensive evaluation of monoclonal antibody production by bioprinted CHO DG44 constructs and highlights how cultivation mode shapes cell growth, metabolism, and productivity in such systems. Across all tested bioproduction scenarios, bioprinted CHO DG44 cells exhibited minimal proliferation compared with the suspension culture, with only a ∼2-fold expansion over 14 days. This restricted growth is most likely driven by the intrinsic limitations of the hydrogel matrix, combining constrained available space and diffusion barriers that generate strong nutrient and oxygen gradients within the constructs. These gradients resulted in a productive peripheral zone limited to approximately the first 250 µm of the constructs, while cells located deeper remained largely nonproliferative and nonproductive.

Metabolically, 3D cultures displayed reduced central carbon metabolism compared with suspension cultures. Semicontinuous feeding promoted continuous glucose consumption and lactate production, whereas batch cultures exhibited metabolic arrest after midrun. Notably, glucose was never depleted, and inhibitory lactate levels were not reached, indicating that nutrient availability in the bulk medium was not the primary growth-limiting factor. These results strongly indicate that increasing the cell-to-medium ratio could significantly improve the efficiency of substrate utilisation and overall process performance.

Monoclonal antibody production closely tracked trends linked to metabolic activity and cultivation mode. While IgG1 mAb titres and space–time yields remained markedly lower than in the classical suspension-fed-batch culture, cell-specific productivities achieved under 3D-amplified semicontinuous conditions were comparable with those obtained in suspension. The introduction of a 3D amplification step systematically doubled cumulative mAb production and space–time yields, highlighting its relevance for improving the productivity of biofabricated systems. Immunohistological analyses further demonstrated that mAb secretion occurs predominantly in the peripheral, proliferative regions of the constructs, confirming the strong coupling between spatial cell organisation, viability, and production.

Altogether, this study demonstrates that 3D bioprinting can support functional monoclonal antibody production by CHO DG44 cells. The current configuration, while being not competitive with established suspension bioprocesses in terms of volumetric productivity, still presents the interesting feature of a comparable cell-specific productivity achieved under optimised 3D conditions. Thus, future developments may focus on (i) increasing the cell-to-medium ratio to exploit available nutrients better and (ii) reducing construct dimensions or implementing more dispersed architectures to alleviate diffusion limitations and suppress nonproductive core regions. Such strategies might unlock the full potential of 3D bioprinting as an innovative and intensifiable platform for mammalian cell-based bioproduction, as it is currently operated in perfusion bioreactor-based scale-up systems (Chastagnier et al., 2026) and has reached printing scales of approximately 75 cm^3^ (Dufour et al., 2024). Even though the scalability of bioprinting is limited compared with stirred tank suspension bioreactors, leveraging mAb-specific productivity using 3D encapsulation could offer encouraging prospects for compact culture systems.

While this study focuses on CHO-DG44 cell behaviour in the 3D culture, CHO-K1 is another widely used host for industrial mAb production (Könitzer et al., 2015; Reinhart et al., 2019). Previous studies have reported differences between the two cell lines in productivity and glycosylation profiles with CHO K1 being more productive. It would therefore be valuable to investigate whether similar 3D culture effects are observed in CHO-K1 cells and how the host–cell background influences the resulting productivity and product quality attributes. Additionally, this study focuses on the production of IgG1, which is the most prevalent on the biopharmaceutical market. However, other isotypes such as IgG4 also exist and show higher viscosity and self-assembly than IgG1, which could have a significant effect on the mAb retention in the hydrogel (Lai et al., 2021). Finally, perspectives include expanding the study to CHO-K1 cells to investigate the effect of the 3D culture on different cell lines and to evaluate the mAb isotype effect on retention in hydrogels.

## Data Availability

The raw data supporting the conclusions of this article will be made available by the authors, without undue reservation.
