# Supplementary material for: 3D-bioprinted microenvironments shape the growth and IgG1 monoclonal antibody production of CHO DG44 cells
Source: Front Bioeng Biotechnol. 2026 Sep 24;14:1854326. doi: 10.3389/fbioe.2026.1854326 (PMC13647993; doi:10.3389/fbioe.2026.1854326)
Supplement: Supplementary file 1 [file Supplementaryfile1.docx]

***Supplementary Information 1***

For all mass to volume conversions, bioink density was assimilated to 1 g/mL in accordance with biomaterial characterisation performed previously (data not shown).

Cell yield after bioprinting was calculated as follows:

$$Y_{printing}=\frac{X_{post-extrusion bioink}}{X_{prior extrusion bioink}}$$

Growth rates were calculated as follows:

$$\mu_{t}=\frac{X_{t}-X_{t_{-1}}}{t-t_{-1}}*\frac{1}{X_{t}}$$

Glucose cumulative consumption was calculated as follows:

$$m_{Glc}= \sum_{i=1}^{n} {(C_{Glc,0}-C}_{Glc,i})*2$$

Lactate cumulative production was calculated as follows:

$$m_{Lac}= \sum_{i=0}^{n} C_{Lac,i}*2$$

mAb cumulative production was calculated as follows:

$$m_{mAb}= \sum_{i=0}^{n} C_{mAb,i}*2$$

Cell specific productivities and consumptions in 3D cultures were calculated as follows:

$$q_{mAb, 3D}=\frac{m_{mAb}}{IVC}$$

$$q_{Glc, 3D}=\frac{m_{Glc}}{IVC}$$

$$q_{Lac, 3D}=\frac{m_{Lac}}{IVC}$$

With:

$$IVC=\int_{t_{0}}^{t} x dt=\frac{x_{1}*\left( t_{0}+t_{1} \right)}{2}+ \sum_{i=1}^{n} \frac{x_{i}*\left( t_{i+1}+t_{i-1} \right)}{2}+\frac{x_{n}*\left( t_{n-1}+t_{n} \right)}{2}$$

Cell specific productivity in suspension culture in phase i was calculated as follows:

$$q_{mAb, i}=\frac{C_{mAb,i}-C_{mAb,i-1}}{{IVCC}_{i}-{IVCC}_{i-1}}$$

$$q_{Glc, i}=\frac{C_{Glc,i}-C_{Glc,i-1}}{{IVCC}_{i}-{IVCC}_{i-1}}$$

$$q_{Lac, i}=\frac{C_{Glc,i}-C_{Glc,i-1}}{{IVCC}_{i}-{IVCC}_{i-1}}$$

With:

$$IVCC=\int_{t_{0}}^{t} x dt=\frac{X_{1}*\left( t_{0}+t_{1} \right)}{2}+ \sum_{i=1}^{n} \frac{X_{i}*\left( t_{i+1}+t_{i-1} \right)}{2}+\frac{X_{n}*\left( t_{n-1}+t_{n} \right)}{2}$$

Metabolic yields were calculated in bioprinted slabs as follows:

$$Y_{X/Glc,3D}= \frac{x_{Max}- x_{0}}{m_{Glc}}$$

$$Y_{Lac/Glc,3D}= \frac{m_{Lac}}{m_{Glc}}$$

Metabolic yields were calculated in suspension culture in Phase i as follows:

$$Y_{X/Glc,i}= \frac{X_{i}- X_{i-1}}{C_{Glc,i}-C_{Glc,i-1}}$$

$$Y_{Lac/Glc,i}= \frac{C_{mAb,i}-C_{mAb,i-1}}{C_{Glc,i}-C_{Glc,i-1}}$$

Space-Time Yields were calculated for 3D cultures as follows:

$${STY}_{3D}= \frac{m_{mAbs}}{V_{total}*t_{culture}}$$

Space-Time Yields suspension fed-batch culture for phase i as follows:

$${STY}_{Fed-batch, i}= \frac{{(C}_{mAbs,i}*V_{i})-{(C}_{mAbs,i-1}*V_{i-1})}{(t_{i}-t_{i-1})*\bar{V_{i-1,i}}}$$

Statistical errors were calculated as follows for different cases; A, B, C, D and E being average values with associated standard errors σ:

| $A=B+C$  $A=B-C$ | $\sigma_{A}=\sqrt{\sigma_{B}^{2}+\sigma_{C}^{2}}$ |
| --- | --- |
| $A=\frac{B}{C}$ | $\sigma_{A}=\left\vert\frac{B}{C} \right\vert\sqrt{\left( \frac{\sigma_{B}}{B} \right)^{2}+\left( \frac{\sigma_{C}}{C} \right)^{2}}$ |
| $A=\frac{B-D}{C}$ | $\sigma_{A}=\left\vert\frac{B-D}{C} \right\vert\sqrt{\frac{\sigma_{B}^{2}+\sigma_{D}^{2}}{{(B-D)}^{2}}+\left( \frac{\sigma_{C}}{C} \right)^{2}}$ |
| $A=\frac{B-D}{C-E}$ | $\sigma_{A}=\left\vert\frac{B-D}{C-E} \right\vert\sqrt{\frac{\sigma_{B}^{2}+\sigma_{D}^{2}}{{(B-D)}^{2}}+\frac{\sigma_{C}^{2}+\sigma_{E}^{2}}{{(C-E)}^{2}}}$ |

List of symbols:

| Symbols |  |
| --- | --- |
| $\boldsymbol{\mu}_{\boldsymbol{t}}$ | Specific growth rate (day^-1^) |
| $\boldsymbol{\mu}_{\boldsymbol{max}}$ | Maximum specific growth rate (day^-1^) |
| t | Time (day) |
| $\boldsymbol{X}_{\boldsymbol{t}}$ | Cell concentration (.10^6^ cells/mL or .10^6^ cells/g of hydrogel) |
| $\boldsymbol{x}_{\boldsymbol{t}}$ | Total cell number in one bioprinted construct (.10^6^ cells) |
| $\boldsymbol{m}_{\boldsymbol{i}}$ | Cumulative mass of metabolite i (mg) |
| $\boldsymbol{C}_{\boldsymbol{i}}$ | Concentration of metabolite I (mg/mL) |
| $\boldsymbol{q}_{\boldsymbol{i}}$ | Specific production of metabolite I (pg.cell^-1^.day^-1^) |
| IVC | Integral Viable Cell number in one bioprinted construct (.10^6^cells.day) |
| IVCC | Integral Viable Cell Concentration (.10^6^ cells.day.mL^-1^) |
| Y_printing_ | Yield of cells recovered from bioprinting (cells.mL^-1^/cells.mL^-1^) |
| Y_X/Glc_ | Number of cells generated per mass of glucose consumed (.10^6^cells/mg) |
| Y_Lac/Glc_ | Mass of lactate produced per mass of glucose consumed (mg/mg) |
| Y_mAb/Glc_ | Mass of antibody produced per mass of glucose consumed (.10^-3^mg/mg) |
| σ | Statistical error of a parameter |

**Supplementary information 2**

Live/Dead Calcein/Ethidium homodimer staining’s at day 1 and day 14 of production phases, red signal represents dead cells, and green signal represents live cells. For live cell staining, bioprinted slabs were placed in 2 mL of a 2 µM calcein-AM (C14-30, Invitrogen) in PBS solution and incubated at 37°C for 30 minutes. Then, dead cell staining is performed through the addition of 1 µL of ethidium homodimer (L3224, Invitrogen) per mL of labelling solution and incubated at 37°C for extra 5 minutes. The slabs were then placed under a 494/517 nm excitation/emission filter for calcein fluorescence imaging and a 523/527 nm excitation/emission filter for Ethidium Homodimer fluorescence imaging. Images were acquired using a NIKON camera with a NIKON SMZ1270 binocular magnifying glass zoom of 10X and a NIKON ECLIPSE Ts2R inverted microscope. Composite images of live and dead cells images were created using ImageJ (Fiji) software.

**Supplementary information 3**

Monoclonal antibody cumulative production in Batch culture scenario in the culture supernatant and in the dissociation supernatant. Quantification of mAbs was done using the same ELISA protocol for both assays and quantity recovered in the dissociated supernatant represents the residual mAbs trapped in the hydrogel.
